# Implementation of a Rural Community Diagnostic Testing Strategy for SARS-CoV-2 in Upstate South Carolina

**DOI:** 10.3389/fpubh.2022.858421

**Published:** 2022-04-05

**Authors:** Emily V. Plumb, Rachel E. Ham, Justin M. Napolitano, Kylie L. King, Theodore J. Swann, Corey A. Kalbaugh, Lior Rennert, Delphine Dean

**Affiliations:** ^1^Research and Education in Disease Diagnosis and Intervention (REDDI) Lab, Center for Innovative Medical Devices and Sensors, Clemson University, Clemson, SC, United States; ^2^Department of Bioengineering, Clemson University, Clemson, SC, United States; ^3^Swann Medicine, Clemson, SC, United States; ^4^Department of Public Health Sciences, Clemson University, Clemson, SC, United States

**Keywords:** SARS-CoV-2, COVID-19, surveillance, rural, community health, saliva testing

## Abstract

By developing a partnership amongst a public university lab, local city government officials and community healthcare providers, we established a drive-through COVID-19 testing site aiming to improve access to SARS-CoV-2 testing in rural Upstate South Carolina. We collected information on symptoms and known exposures of individuals seeking testing to determine the number of pre- or asymptomatic individuals. We completed 71,102 SARS-CoV-2 tests in the community between December 2020-December 2021 and reported 91.49% of results within 24 h. We successfully identified 5,244 positive tests; 73.36% of these tests originated from individuals who did not report symptoms. Finally, we identified high transmission levels during two major surges and compared test positivity rates of the local and regional communities. Importantly, the local community had significantly lower test positivity rates than the regional community throughout 2021 (*p* < 0.001). While both communities reached peak case load and test positivity near the same time, the local community returned to moderate transmission as indicated by positivity 4 weeks before the regional community. Our university lab facilitated easy testing with fast turnaround times, which encouraged voluntary testing and helped identify a large number of non-symptomatic cases. Finding the balance of simplicity, accessibility, and community trust was vital to the success of our widespread community testing program for SARS-CoV-2.

## Introduction

As of December 1, 2021, South Carolina has identified over 890,000 COVID-19 cases, despite limited testing sites (1). Because a large population of infected, contagious individuals are asymptomatic or pre-symptomatic, readily accessible testing is essential to identify and isolate these individuals (2). Testing frequency and turnaround time are critical components to reducing outbreaks regardless of testing method (3, 4); however, supply chain shortages early on in the pandemic limited widespread testing (5, 6). Testing capacity was also exacerbated in rural areas with less access to testing centers and clinical laboratories (7–9). In the following report, we describe the development of a robust community testing strategy in rural Upstate SC.

The number of patients seeking diagnostic testing outpaced healthcare personnel availability as the pandemic escalated. At the start of the pandemic, our community relied primarily on molecular-based testing *via* nasopharyngeal swabs conducted through the South Carolina Department of Health and Environmental Control (SCDHEC), and qualified healthcare personnel contacted each patient that tested positive. Transportation distance and slow laboratory processing resulted in long turnaround times, while long lines and unpleasant testing methods discouraged non-symptomatic individuals from testing voluntarily. During high case numbers from COVID-19 surges, testing supply shortages were common in South Carolina^1^ and testing cost upwards of $130^2^.

In response to these growing concerns, we (DD, TJS) established a community partnership between the City of Clemson, SCDHEC, local healthcare practitioners, and the newly opened Research and Education in Disease Diagnosis and Intervention (REDDI) Laboratory at Clemson University (CLIA ID: 42D2193465). Beginning in December 2020, the REDDI Lab offered free molecular-based saliva tests for members of the surrounding community, made possible through funds from the South Carolina Governor's and Joint Bond Review Committee (10). Clemson City elected officials assisted with testing site management and publicity. Tests were registered and reported through a third-party healthcare cloud-based management system, local medical professionals volunteered to contact community members positive for SARS-CoV-2, and DHEC managed contact tracing. With these collaborative efforts, we drastically improved the SARS-CoV-2 diagnostic testing infrastructure for Upstate South Carolina.

Here, we describe the positive effects of improved access to free, efficient COVID-19 diagnostic testing. Between December 1, 2020 and November 30, 2021, we performed 71,102 SARS-CoV-2 tests for the non-university associated Upstate SC community. Proximity to the laboratory along with rapid processing yielded short turnaround times for patient results, with 74.67% reported same-day and 91.49% reported within 24 h. Of the positive cases identified, 73.36% were from pre- or asymptomatic individuals, who may have gone untested without access to our free testing program. We hope to provide a model for public health emergency responses, particularly in rural areas near state-funded research facilities.

## Context

Upstate SC comprises 1.5 million residents; our lab primarily provided testing to Pickens (location of Clemson University), Oconee, Anderson, and Greenville Counties. We defined our local community as three individual towns within 5 miles of the university: Clemson, Pendleton, and Central, with a permanent population of 24,950. The regional community encompasses the remainder of municipalities within all eleven counties (11); a small portion of patients were not residents of Upstate SC and fell into neither group. The Upstate population is 3.9% Latino or Hispanic, 8.5% Black, and 2.4% Asian (12).

We obtained total regional SARS-CoV-2 testing information from SC DHEC for analysis. We identified low testing rates in rural counties such as Pickens and Oconee prior to the establishment of the REDDI Lab in December 2020. In addition to providing community testing access, the REDDI Lab performed weekly surveillance testing of the entire university population, including faculty and staff (13).

## Key Programmatic Details

### Community Site Design

**Figure 1:**
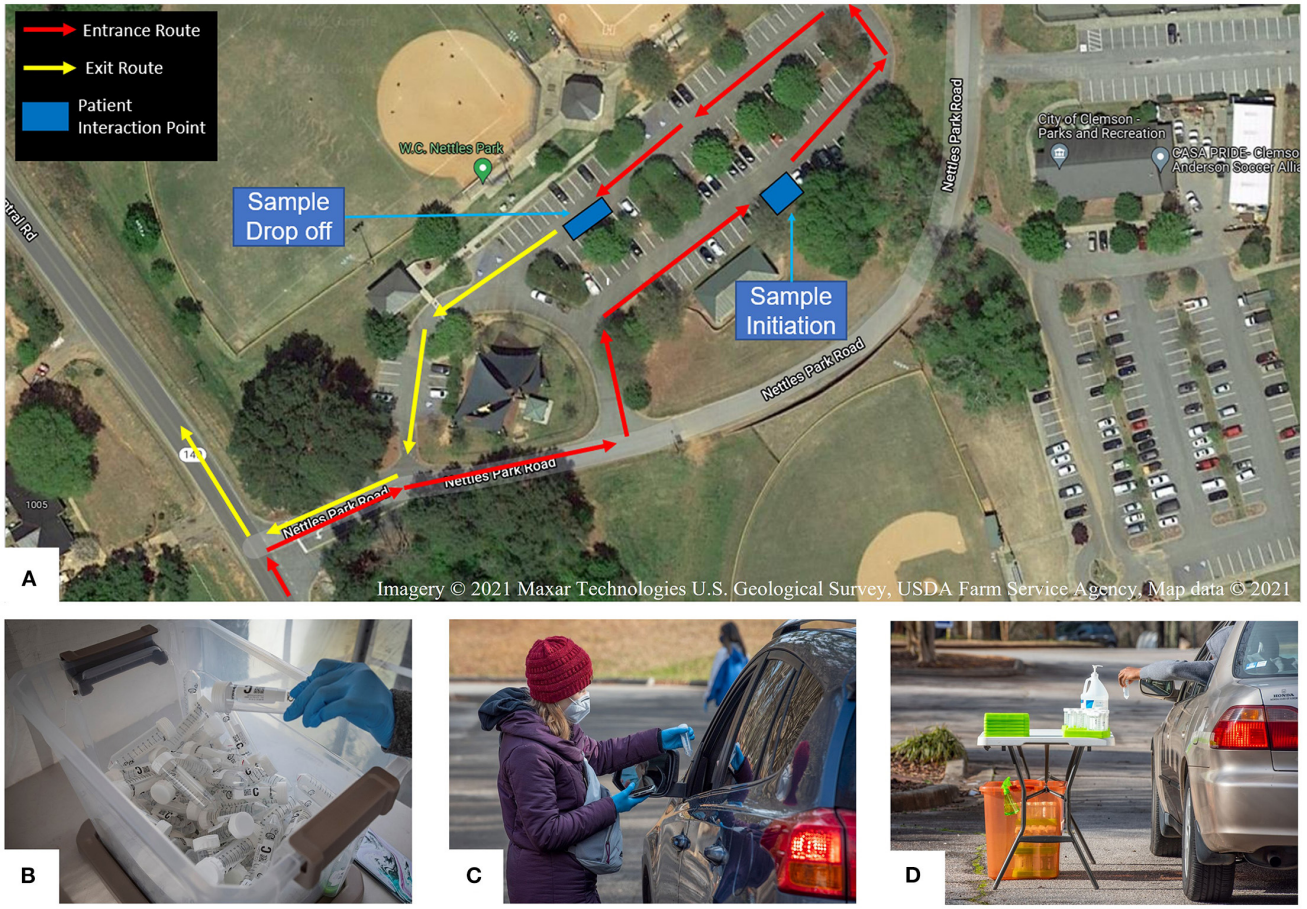
REDDI Laboratory community testing operation. **(A)** Overhead diagram of the community testing site layout. **(B)** Box of 50 ml saliva collection tubes labeled with unique QR codes. **(C)** Collection staff initiating a test for a community member. **(D)** Community member placing self-collected sample into a collection tray. Filled sample trays were placed in the orange biohazard box for transportation to the laboratory.

In July 2020, we began to explore options to offer free community SARS-CoV-2 testing and considered collection at medical offices, permanent community locations, and mobile clinics. In the early stages, local medical practices collected samples from symptomatic patients, but this method was not accessible to the general public. We opted for a permanent community testing location for ease of set-up and communication. Publicly owned spaces were evaluated as testing venues using three criteria: travel distance to the performing laboratory, parking lot space, and safety for testing personnel. Initially, we utilized the local fire station for the testing site; however, space was limited, hindering efforts to scale up testing. Our solution was to shift the testing site to a public park (Nettles Park) three miles from the laboratory. This park offered large, paved areas appropriate for larger scale drive-up testing (Figure 1A).

Starting in January 2021, we utilized mobile clinic testing sponsored through the Healthy Me, Healthy SC collaborative. Community leaders from churches and local governments in Pickens and Oconee initiated tests and the mobile clinic made weekly trips to collect the samples. After we expanded hours at the Nettles Park site, we found most community members preferred to visit the site rather than the mobile clinic due to greater flexibility.

### Testing Process

Test materials were assembled at the performing laboratory and included specimen trays, biohazard bags, and 50 ml conical tubes labeled with unique QR codes (Figure 1B). The supply chain of materials was maintained by laboratory staff, and courier personnel replenished materials when delivering specimens from the testing location.

We utilized a third-party HIPAA-compliant cloud-based reporting platform (Rymedi, version 1.0, Greenville SC, USA) to reduce the burden of patient registration and record keeping. Each patient was required to fill out personal health information through the online interface to generate a patient identification QR code. Patients were encouraged to register prior to arrival at the testing site; however, onsite registration was also available. After initial registration, the patient could reuse the same personal QR code for subsequent tests.

In order to initiate an individual test, collection staff scanned both the patient QR code and the QR code on the collection tube to pair the information. QR codes were scanned with either industry standard barcode readers or phone cameras. Collection staff also asked patients about any symptoms and/or recent exposures and recorded tests as either Symptomatic, Exposed, or Standard. Patients received an email and a text message confirming that their test was registered in the system. To maximize efficiency at the testing site, we implemented a drive-thru system with two stations: one for staff to confirm patient information and initiate tests, and a second for patients to deposit specimen tubes into collection trays (Figures 1C,D).

We chose saliva-based testing due to convenience of sample collection; patients could self-collect samples, minimizing transmission risk to personnel and other individuals at the site (14, 15). The saliva RT-qPCR diagnostic test that we used is a modified version of the EUA-approved SalivaDirect protocol (16). We found our protocol to have a 90% sensitivity and 99% specificity when compared to paired nasopharyngeal swab RT-qPCR tests (17). Several studies have also shown that saliva qPCR COVID-19 diagnostic tests may even be more accurate than anterior nasal swab tests (18). Patients were asked to avoid eating or drinking for 30 min prior to arrival. After registering the kit, collection staff would instruct patients to collect 1 ml of saliva in the 50 ml collection tube, then place their tube on a specimen collection tray. Samples were transported in batches to the lab at the end of each daily collection.

A blanket standing physician order allowed testing for all presenting patients, regardless of symptoms, provider orders, or proof of insurance. All adults and children above the age of 6 could obtain testing at the site. Infants and younger children were permitted to attempt the test, however, it can be difficult for this age group to produce saliva on demand.

Once the specimens were processed at the laboratory, the test results were posted to the reporting platform and patients were notified *via* email and text message that the results were available. Actual results were available through a weblink to the individual account. We organized a community network of volunteer medical professionals to download result files from the online platform and contact individuals that tested positive within 24 h of their results. The participants who tested positive received up-to-date information from the CDC and SCDHEC about quarantine and isolation guidelines. At the beginning of our community site, CDC guidance recommended a 14-day isolation period. This was shortened to 10-day isolation in January 2021 until the end of the study period. Participants were not required to obtain a negative test to exit isolation. Contact tracing was carried out by SCDHEC.

It should be noted that during follow-up calls to positive patients by the medical volunteers, several patients, who had Exposed or Standard tagged tests, did admit to having symptoms at the time of testing. When asked why they did not report symptoms at the test site, the majority of patients said they did not realize their symptoms were related to COVID-19. In response, our team increased outreach projects to educate community members on COVID-19 symptoms through social media posts, posts on the Clemson city hall website, community panel discussion sessions, and flyers.

The community testing site was open every weekday morning to allow for same-day laboratory processing. However, many population cohorts were unable to travel to the testing site at these times, such as preschool teachers, shift workers, and stepped-care neighborhood residents. To overcome these barriers, we taught community members how to initiate tests and arranged courier services to deliver the specimens to the laboratory. For example, a school administrator could initiate tests independently and provide saliva collection instructions to school staff. These samples could then be delivered to the lab at a convenient time.

### Outreach

To increase awareness and participation in community testing, we invited newspapers and local TV stations to tour the lab and interview the staff. We partnered with Clemson City elected officials to update community testing information on the municipal website and to post flyers at the free community clinic. We also posted large yard signs near the testing site to increase visibility. In addition, we maintained a social media presence and hosted question and answer sessions with the lab directors about COVID-19 symptoms, testing, vaccines, and impact of variants.

### Statistical Analysis

Pearson's chi-squared tests with Yates continuity correction were performed between the local and regional groups found in **Figure 3B** and between the standard, exposed, and symptomatic groups found in **Figure 5**. These tests were performed across the entire duration of the study and at specific time intervals, and all returned p-values of < < 0.001.

## Results

**Figure 2:**
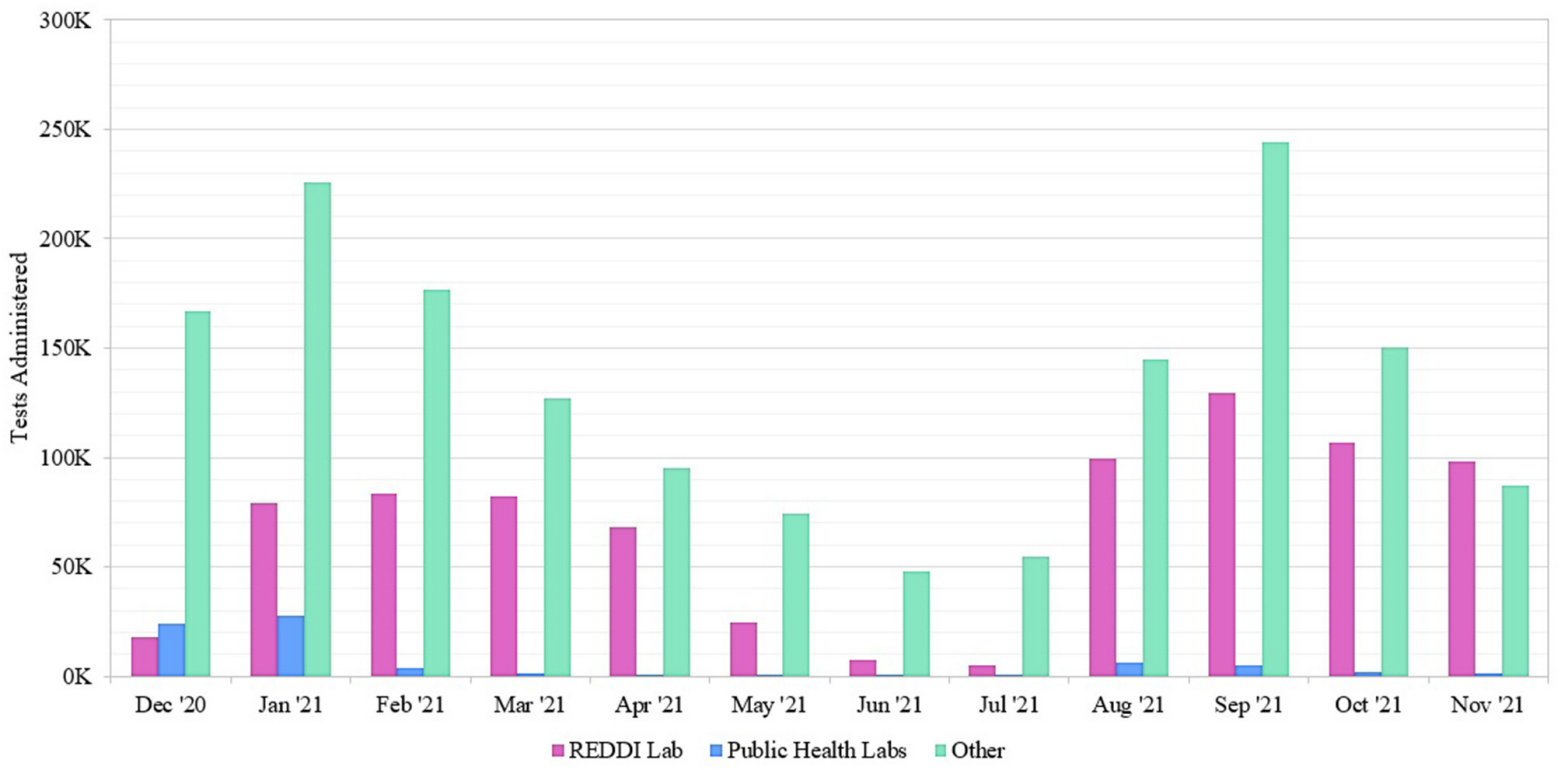
Number of SARS-CoV-2 molecular tests performed at Upstate South Carolina testing sites offered by SCDHEC, REDDI Lab, and other labs from December 1, 2020–November 30, 2021. The REDDI Lab group includes all community and non-community molecular tests performed by the REDDI Lab.

To determine the impact of REDDI Lab community testing, we gathered data from SCDHEC regarding molecular testing options across Upstate South Carolina from December 1, 2020–December 1, 2021 (Figure 2). The “other” options included commercial labs and hospital clinic labs not sponsored by the state of South Carolina (1). Our lab was one of the few public university laboratories founded for and dedicated to SARS-CoV-2 diagnostic testing in the Southeast US. SCDHEC public labs encompassed five regional laboratory locations. During this timeframe, the REDDI Lab conducted over 800,000 total tests, including 71,102 community tests, accounting for approximately one-third of the total molecular tests in Upstate SC. By November 2021, we performed the majority of molecular tests in the region. Of all the tests, 0.5% resulted as inconclusive. Most inconclusive tests occurred because the sample did not produce an adequate human control signal due to poor sample quality, as detailed in our methods paper (17). There were no differences between the local and regional inconclusive rates.

**Figure 3:**
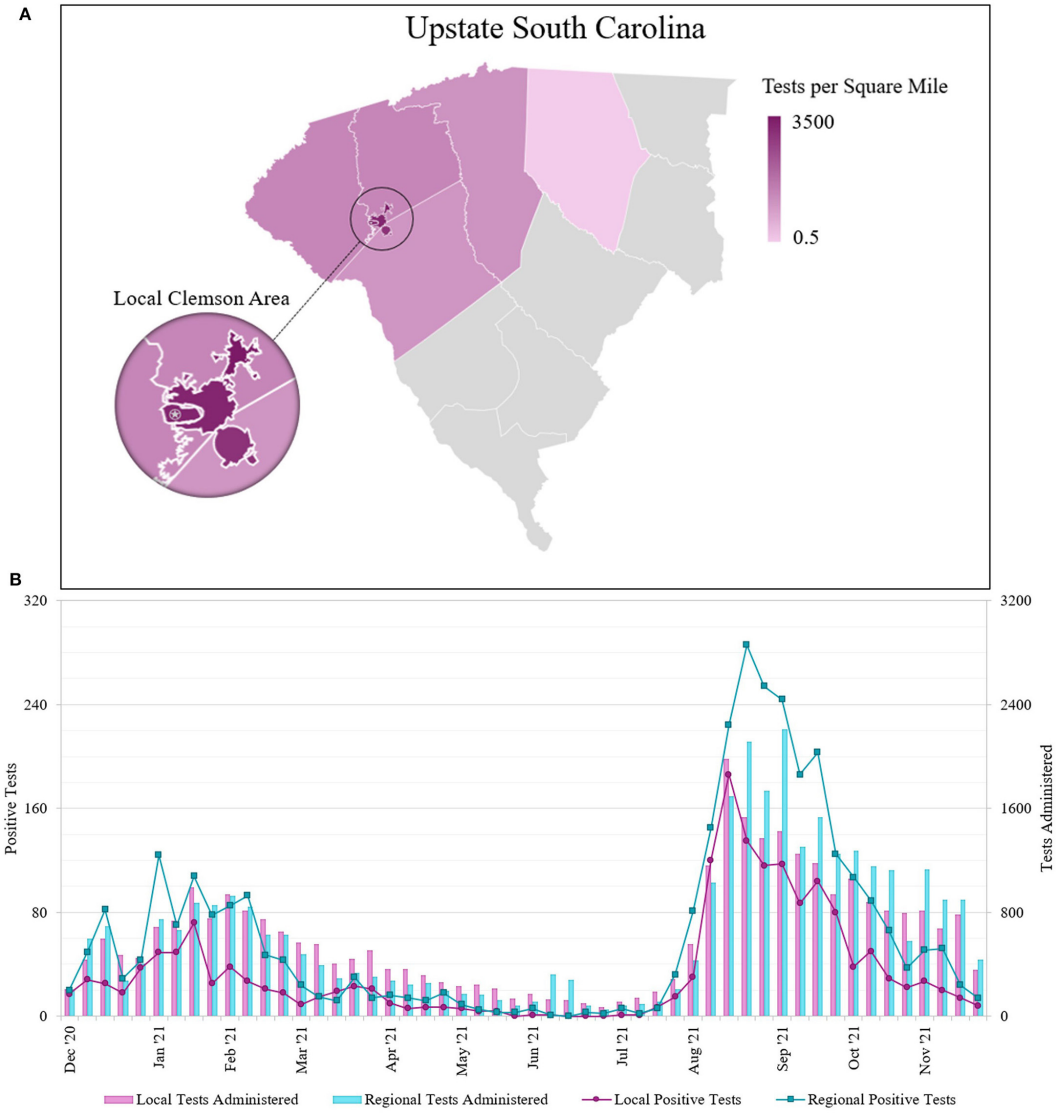
**(A)** Geographical distribution of SARS-CoV-2 molecular tests performed at the REDDI Lab from December 1, 2020–November 30, 2021 across Upstate SC. Nearly half of all REDDI community tests performed in Upstate SC were performed for residents of local towns of Central, Clemson, and Pendleton. The star indicates the REDDI Lab. Data is presented in tests per square mile. Tests in local towns were subtracted from the county totals and local town areas were subtracted from county areas; counties with 500 tests or less were omitted. The municipalities of Clemson and Clemson University were combined for community tests totals. **(B)** Number of SARS-CoV-2 saliva tests administered and positive results sorted by patient ZIP code. Local area is defined as the three nearest municipalities: Clemson, Central, and Pendleton (ZIP codes 29631, 29632, 29633, 29634, 29630, and 29670). The regional area includes all other municipalities in Upstate South Carolina, including those omitted from 3A. Note that the positive test number and test administered axes are scaled such that the positive test axis is 10% of the test administered axis.

We sorted tests by participant ZIP code to determine local and regional community testing participation (Figure 3A). We defined the local community as ZIP codes 29630, 29631, 29632, 29633, 29634, and 29670. All other Upstate SC residents were considered the regional community. Local community members accounted for 31,992 tests and regional community members accounted for 33,797 tests. There were 5,313 tests that did not fall into either of the categories; we excluded those who were not residents of Upstate SC and those who had invalid ZIP codes on file. We determined the testing density of both communities of tests performed at our lab. We performed an average of 2156.5 tests/sq mile for the local community, and an average of 11.3 tests/sq mile for the regional community.

We also assessed the number of positive cases for individuals from different geographical areas seeking community testing (Figure 3B). We identified two outbreaks spanning January-February 2021 and August-September 2021. From sequencing data, we determined that while the winter (Jan-Feb 2021) outbreak contained a mix of different strains (primarily Alpha and Gamma), the summer (Aug-Sep 2021) was primarily driven by the Delta variant of concern (19–21). Across the entire study duration, we found statistically significant differences (*p* < 0.001) in the positivity rates between the local and regional communities. We found that the average percent positivity rate for the local community was 5.57%, while the regional community was 9.86%. During the winter outbreak, the local community average percent positivity rate was found to be 4.79% as opposed to 10.61% in regional community; lastly, during the summer outbreak, the local community average positivity rate was 8.65% as opposed to 13.33% in the regional community. The World Health Organization (WHO) public health policy classifies community transmission levels by positivity rate: moderate transmission is 2–5%, high transmission is 5–20%, and extremely high transmission is >20% (22). During the winter outbreak, the local community only exceeded moderate community transmission (<5% positivity) for 3 weeks, while the regional community remained at high community transmission (5–20% positivity) for 9 weeks. Additionally, the local community maintained moderate transmission for 25 of the 30 weeks in 2021 prior to the summer outbreak, while the regional community did so for merely 12 of the 30 weeks. After the Delta surge, the local community returned to moderate transmission for 7 of 8 remaining weeks, while the regional community only returned to moderate transmission for 3 of 8 remaining weeks.

**Figure 4:**
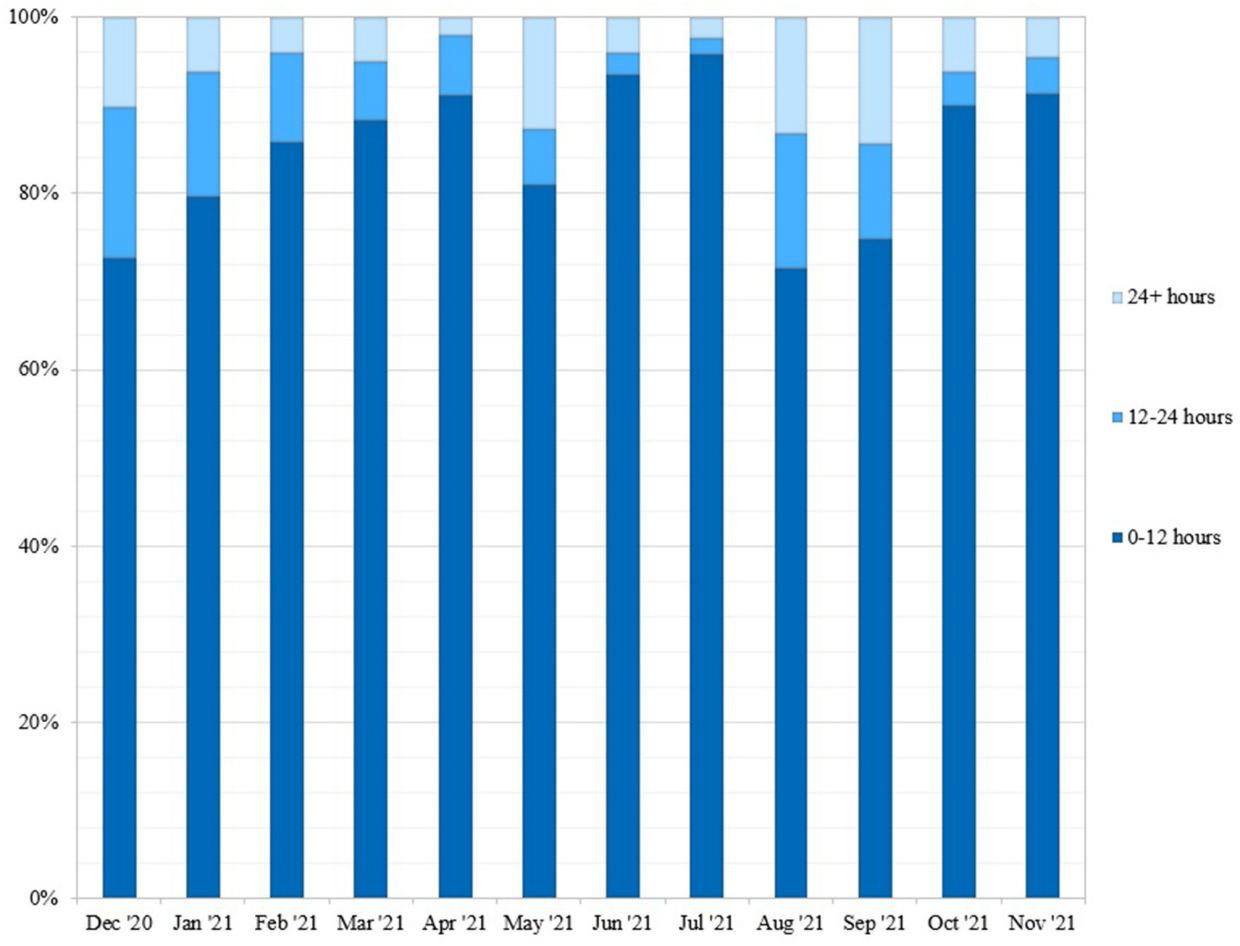
Weekly turnaround time for community tests performed at REDDI Lab from December 1, 2020-November 30, 2021. Turnaround time was calculated by taking the difference between time of sample collection and time of result. This data was obtained from the clinical reporting platform (Rymedi, version 1.0, Greenville SC, USA).

Next, we determined the turnaround time for community tests between December 1, 2020–November 30, 2021 (Figure 4). 74.67% of tests were reported on the same day, with 91.49% of tests completed within 24 h of saliva collection. For comparison, we gathered turnaround time data from other molecular testing locations in Upstate SC, including Lowcountry Urgent Care, SCDHEC, TourHealth, CVS Pharmacy, American Family Care Urgent Care, and the Medical University of South Carolina Health (1). All testing organizations reported average result turnaround times between 24 and 48 h or greater. While some labs report turnaround time starting at time of lab accession, all of these COVID-19 test services report turnaround time starting at time of test initiation.

**Figure 5:**
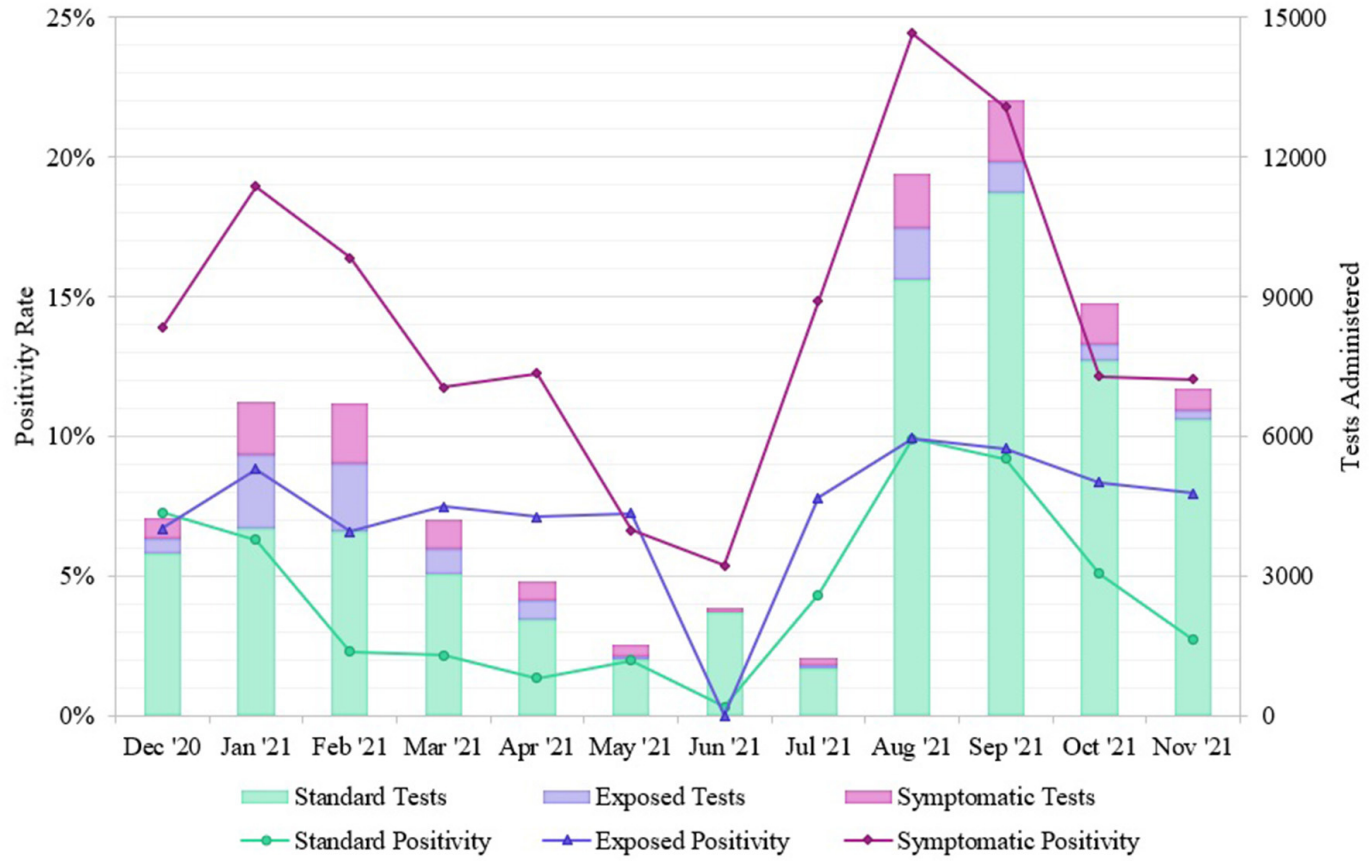
Positivity rates of SARS-CoV-2 tests from different REDDI Lab community testing groups from December 1, 2020–November 30, 2021. Individuals self-reported exposures or symptoms during test initiation, so this can only serve as an approximate measurement. These data were obtained from the clinical reporting platform (Rymedi, version 1.0, Greenville SC, USA).

We separated the positivity rates by Standard, Exposed, and Symptomatic testing groups to examine the prevalence of non-symptomatic cases (Figure 5). Exposed and Standard testing individuals did not report symptoms at the time of testing. Non-symptomatic individuals accounted for 88.32% of total tests (78.72%, Standard, and 9.60%, Exposed), and 73.36% of positive tests originated from these groups (62.76%, Standard, and 10.60%, Exposed). The overall positivity rates for Standard, Exposed, and Symptomatic groups were 5.99%, 8.27%, and 17.13%, respectively. During the winter outbreak, positivity rates for Standard, Exposed, and Symptomatic were 4.39%, 7.82%, and 17.72%, respectively; during the summer outbreak Standard, Exposed, and Symptomatic rates were 9.63%, 9.90%, and 23.45%, respectively. During non-outbreak periods, the Standard, Exposed, and Symptomatic rates were 3.69%, 7.47%, and 11.98%, respectively.

## Discussion

We performed about 2,000 tests/sq mile in the local community, while we only performed about 10 tests/sq mile in the regional community (Figure 3A). Although we administered a comparable total of diagnostic tests for local and regional community members throughout 2021, the positive case count remained consistently lower in the local area (Figure 3B). Furthermore, positivity rates were significantly different between the local and regional communities (*p* < < 0.001) throughout the timeframe. Because the local community has a smaller population than the region, testing rates may have approached adequate surveillance and provided a buffer against a severe outbreak (23).

The REDDI Lab garnered a positive reputation for fast, convenient, and free testing at a consistent location with a predictable schedule. We reported same-day results for 74.67% of tests and results within 24 h for 91.49% of tests, even as the daily testing capacity increased dramatically (Figure 4). Our rapid turnaround time encouraged voluntary testing and provided actionable information for clinicians to report to patients. Same-day results also allowed community members to self-isolate and seek treatment in a timelier manner, which may have decreased transmission. In addition, some businesses and schools in Upstate SC required a negative test to return to in-person participation post-infection. The CDC recommended exposed individuals to quarantine for 10 days if no symptoms were present, or 7 days if the individual received a negative test result. Patient feedback indicated that many used our testing service for their quarantine exit test and to fulfill travel requirements. In addition, while we were not able to do formal surveys of our patient population, feedback from patients at our community outreach events and comments from positive patients during the medical follow up call indicated that some patients would not have tested at all had our test site not been available. Deterrents to other testing options mentioned in these comments were accessibility to testing sites and apprehension of nasopharyngeal/nasal tests.

Although the symptomatic group had a higher positivity rate throughout the timeframe, test positivity among patients who did not report symptoms at the time of testing was still quite high, particularly during periods of high community transmission and exposure (Figure 5). During the winter outbreak spanning Jan-Feb 2021, individuals reporting exposure had a 7.82% average positivity rate while individuals seeking standard testing remained at 4.39% positivity, indicating that community members were accurate at determining their own exposures. The total positivity rate decreased to 2.23% by April 30, 2021, indicating that this outbreak had fully resolved, and community prevalence was low. During the summer outbreak from Aug-Sep 2021, both non-symptomatic groups had similar positivity rates (Standard, 9.63% and Exposed, 9.90%), indicating that all community members likely experienced exposures in daily life. This trend matches observations from other communities; the majority of these infections were caused by the Delta variant (19) as confirmed by whole genome sequencing of our samples (20, 21), which has been linked to higher levels of community transmission (24). After the Delta surge, the positivity rate for the standard testing population returned to 4.08% while the exposed positivity rate remained at 8.33%, indicating a decrease in community transmission. Additionally, 73.36% of the positive cases we identified originated from non-symptomatic individuals. Studies indicate that the viral load, and thereby infectious potential, is similar in both asymptomatic and symptomatic patients (25). This is consistent with the Ct values observed from the diagnostic saliva qPCR test run in our lab on symptomatic vs. non-symptomatic patients. Moreover, pre- or asymptomatic spread is a significant driver of transmission in high-contact community settings (26). Therefore, routine surveillance is of the utmost importance to identify non-symptomatic but infectious individuals.

One of the advantages of using saliva is that repeated surveillance testing of non-symptomatic individuals is possible; it can be difficult for patients to tolerate repeated nasopharyngeal swabs. Frequency tolerability is an often-cited advantage of antigen-based testing over RT-PCR for surveillance, along with cost of the test (4, 27). Our saliva test is also low-cost ($4-6 per test including labor and reagents) as it does not require specialized collection devices or extraction reagents (13, 17). Additionally, saliva RT-PCR is more accurate than nasopharyngeal swabs or antigen testing early in the course of infection (28, 29). This made it preferable and affordable for the university administration, local, and state government to support a free saliva test site that was used for frequent repeated testing by patients.

Because we performed our study retrospectively, we do not have information on vaccination rates for the patients utilizing our site. However, according to SCDHEC, the vaccination rates in the Upstate SC counties were well below 50% for most of the time period. In addition, except for Greenville County (part of the regional community), all the vaccination rates in the local and regional counties were within 2% of each other during the entire year. Greenville County vaccination rates were consistently 5–10% higher than other Upstate SC counties. The vaccine rates in the region were well below 50%, even during the Delta surge in August 2021 when vaccines were readily available. Therefore, we do not believe regional variation in vaccination rates had a significant impact on our data.

Throughout our study, mask ordinances for local and regional cities and municipalities changed multiple times. While the exact start and end date of any mask mandate varied by city/region, overall durations and stringency of masking mandates were similar across Upstate SC during this study period. In addition, in Summer 2021, the SC legislature passed several statewide bans on mask, vaccine, and testing mandates, particularly in K-12 schools. Therefore, mask wearing was done by personal choice during most of the study period, which has been shown to be very consistent over the entire Upstate SC region (30). Prior studies have shown that compliance with mask-wearing and other risk mitigation behaviors vary by many factors including age group (31) and mandates (30); however, geographic region was not a significant factor (32).

This report describes the implementation of a COVID-19 testing strategy in a rural community. Utilizing saliva-based testing allowed for safer and easier site operation and allowed for off-site self-testing by community members. Community physicians endorsed testing for screening purposes, and a standing order allowed testing for anyone including individuals under 18 years. The permanent testing site was more advantageous than our mobile testing unit in a rural area with low population density, though access was limited to those who had transportation and could be alleviated in the future. The REDDI Lab facilitated easy testing with fast turnaround times, which encouraged voluntary testing and helped identify a large number of non-symptomatic cases. Finding the balance of simplicity, accessibility, and community trust promoted voluntary participation to help accomplish widespread community screening. Our strategy can inform public policy as a non-restrictive and non-invasive surveillance method. Thus, we hope to serve as a model for other rural communities seeking to apply mitigation strategies in future public health crises.

## Data Availability Statement

The datasets presented in this study can be found in online repositories. The names of the repository/repositories and accession number(s) can be found in the article/Supplementary Material.

## Ethics Statement

Ethical review for this study was obtained by the Institutional Review Board of Clemson University. This is a retrospective study on archived deidentified samples and data; all clinical diagnostics data and samples were stripped of any patient identifiers prior to any analysis performed in this study including any analysis of testing results or trends and sequencing of SARS-CoV-2 for variant identification.

## Author Contributions

JN, TS, and DD contributed to original conception of the paper. EP and RH drafted the manuscript. JN organized the database and created the figures. EP, RH, JN, KK, and DD contributed to analysis and close editing of the manuscript. CK and LR contributed to formal analysis of the manuscript. All authors contributed to, read, and approved the submitted manuscript.

## Funding

The project was funded by SC Governor and Joint Bond Review Committee and NIH NIGMS 3P20GM121342-03S1 (DD).

## Conflict of Interest

The authors declare that the research was conducted in the absence of any commercial or financial relationships that could be construed as a potential conflict of interest.

## Publisher's Note

All claims expressed in this article are solely those of the authors and do not necessarily represent those of their affiliated organizations, or those of the publisher, the editors and the reviewers. Any product that may be evaluated in this article, or claim that may be made by its manufacturer, is not guaranteed or endorsed by the publisher.

## Footnotes

^1^Although this data has not be consolidated to our knowledge, this news article articulates supply chain issues in South Carolina. https://www.reuters.com/world/us/us-covid-19-tests-again-short-supply-infections-soar-schools-reopen-2021-08-27/ (accessed November 20, 2021).

^2^Although cost of COVID-19 tests varies across states and time, this news article mentions high costs to South Carolinians. https://www.reuters.com/business/healthcare-pharmaceuticals/rapid-covid-19-tests-increasingly-scarce-pricey-demand-employers-jumps-2021-10-05/ (accessed October 26, 2021).
