# Supplementary material for: Implementation of a Rural Community Diagnostic Testing Strategy for SARS-CoV-2 in Upstate South Carolina
Source: Front Public Health. 2022 Apr 5;10:858421. doi: 10.3389/fpubh.2022.858421 (PMC9016164; doi:10.3389/fpubh.2022.858421)
Supplement: Supplementary file 1 [file Data_Sheet_1.pdf]

Supplementary Material

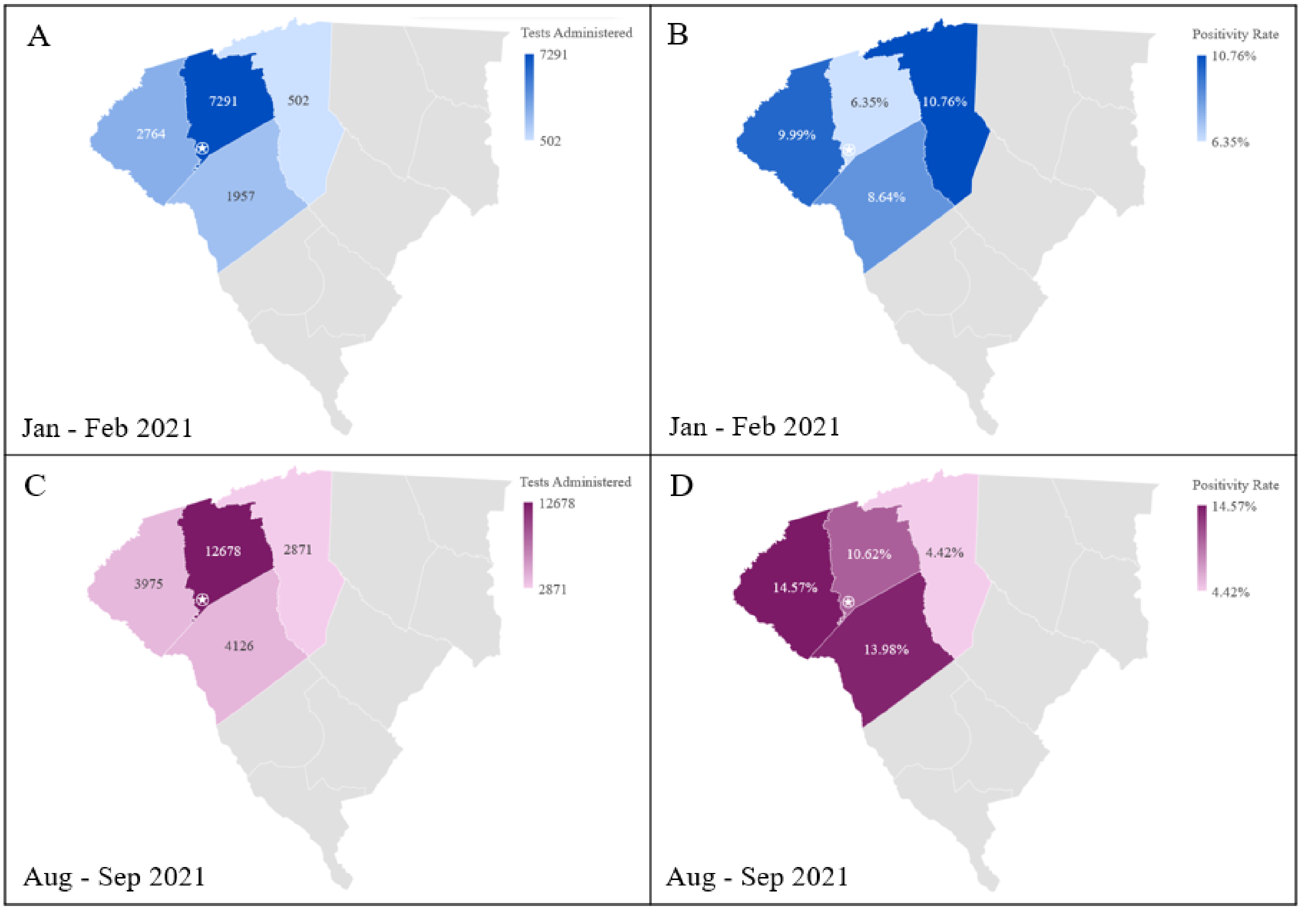

Supplementary Figure 1. Relationship between positivity rate/testing quantity and geographic distribution across Upstate South Carolina during two SARS-CoV-2 outbreaks. Only Pickens, Oconee, Anderson, and Greenville Counties met a minimum number of tests (500) appropriate for analysis. The star indicates Clemson University. The top two panels display the number of tests administered by the REDDI Laboratory **(A)** and the percentage of positive cases **(B)** throughout Jan-Feb 2021. The bottom two panels display the number of tests administered by the REDDI Lab **(C)** and the percentage of positive cases **(D)** throughout Aug-Sept 2021. The changing strain composition during these outbreaks may have contributed to variations in disease prevalence.

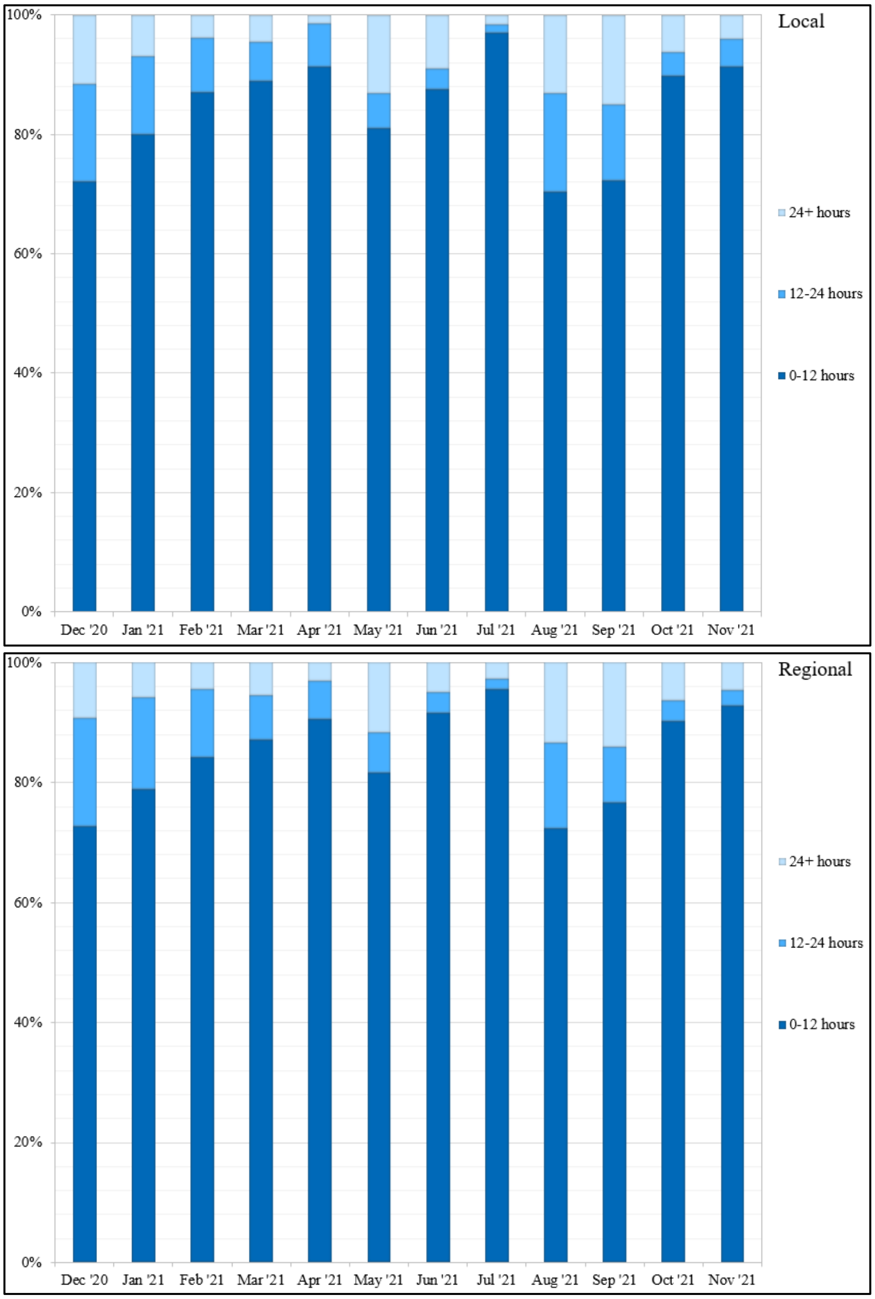

Supplementary Figure 2. Turn-around time for the Local and Regional Community groups. Turn around time was similar between the two groups.
